# Development of a Lipidomics-Based Cell Screening Platform for Indirect Antioxidants Targeting Oxidized Lipid Droplet Formation and Mitochondrial Membrane Abnormality

**DOI:** 10.3390/nu18050719

**Published:** 2026-02-24

**Authors:** Yuzu Shibata, Toshihiro Sakurai, Akiko Sakurai, Misuzu Sato, Shu-Ping Hui

**Affiliations:** Faculty of Health Sciences, Hokkaido University, Sapporo 060-0812, Japan

**Keywords:** metabolic dysfunction-associated steatotic liver disease, oxidized low-density lipoprotein, cardiolipin

## Abstract

**Background/Objectives**: Oxidized lipid droplet formation and cardiolipin (CL) profile abnormality in mitochondrial membranes are associated with metabolic dysfunction-associated steatotic liver disease (MASLD). However, studies on cell models to easily and simultaneously assess the preventive effects on oxidized lipid droplet formation and CL abnormality by indirect antioxidants are limited. Here, we aimed to develop a lipidomics-based cell screening platform to simultaneously evaluate the preventive effects of indirect antioxidants on oxidized lipid droplet formation and mitochondrial membrane lipid abnormalities. **Methods**: We created a novel lipidomics-based cell screening platform using oxidized low-density lipoprotein (oxLDL) and a human liver-derived cell line (C3A), and screened indirect antioxidants to promote the expression of cellular antioxidant enzymes, preventing oxidized lipid droplet formation. **Results**: Mass spectrometry revealed that oxLDL increased the levels of cholesteryl ester hydroperoxides. Thus, oxidized lipid droplet formation was confirmed. Three indirect antioxidants (kaempferol, quercetin, and hesperetin) were examined in the lipidomics-based platform. Consequently, quercetin significantly decreased major lipids and lipid hydroperoxide species, particularly triglycerides and triglyceride hydroperoxides with five or more double bonds. Furthermore, fluorescence microscopy revealed that quercetin reduced the size of small oxidized lipid droplets; it also decreased monolysocardiolipin, which could be associated with mitochondrial dysfunction. **Conclusions**: Overall, we demonstrated that this method could be useful for screening indirect antioxidants with excellent preventive effects against oxidized lipid droplet formation and CL abnormality by simultaneously analyzing various lipids.

## 1. Introduction

Metabolic dysfunction-associated steatotic liver disease (MASLD) is a fatty liver disease caused by metabolic disorders, such as overweight/obesity and type 2 diabetes. Oxidative stress induces inflammation and fibrosis in the livers of patients with MASLD and contributes to the development of metabolic dysfunction-associated steatohepatitis (MASH), which can progress to cirrhosis, liver cancer, and liver failure [1]. The estimated prevalence of MASLD in adults is 38% [2]. This prevalence continues to increase because of the rising prevalence of obesity and type 2 diabetes [3]. In June 2023, the definition/name of nonalcoholic fatty liver disease and nonalcoholic steatohepatitis (NAFLD/NASH) was changed to MASLD/MASH [4]. However, diagnosing MASH requires an invasive liver biopsy. Furthermore, no blood biomarkers for differentiation nor clinically effective treatments have been identified. Given these circumstances, it is important to develop methods to prevent disease onset.

Lipid peroxidation is reportedly higher in the livers of patients with NASH than in those with simple steatosis [5]. Our laboratory previously showed that intravenous administration of oxidized low-density lipoprotein (oxLDL) into mice fed a high-fat diet induces an increase in lipid hydroperoxides in the fatty liver, leading to worsening liver inflammation and fibrosis [6,7]. Moreover, an *in vitro* study revealed that stimulation with oxLDL in a human liver-derived cell line (C3A) increased lipid hydroperoxides and induced the formation of oxidized lipid droplets [8]. Therefore, oxLDL may be involved in the formation of oxidized lipid droplets and contribute to the pathogenesis of MASLD/MASH. In addition, reducing oxidized lipid accumulation in hepatic lipid droplets using antioxidants could be useful in preventing the onset of MASLD/MASH. Indirect antioxidants promote the expression of intracellular antioxidant enzymes via the Keap1-Nrf2 pathway [9] and provide more sustained cytoprotection than direct antioxidants [10]. Kaempferol, quercetin, and hesperetin are common flavonoids with similar chemical structures that activate the Keap1-Nrf2 pathway, leading to the expression of antioxidant enzymes [11,12,13]. Flavonoids are commonly consumed food components [14]. Kaempferol, quercetin, and hesperetin are abundant in tea leaves and kale, onions, apples, lemons, and yuzu, respectively. Furthermore, absorbed polyphenols are converted not only into aglycones but also into metabolites, such as conjugates, in the bloodstream [15,16,17]. Oral ingestion is known to increase circulating levels of aglycones and conjugates. These metabolites are taken up into cells, where conjugates are cleaved by enzymes such as β-glucuronidase and are therefore thought to function as aglycones [18].

However, few lipidomics-based studies have focused on cell models and target molecules to easily assess the preventive effects of indirect antioxidants on oxidized lipid droplet formation. To establish a clearer experimental system, this study focused on flavonoid aglycones.

Structural and functional abnormalities have been found in the liver mitochondria in MASLD [19]. Cardiolipin (CL), a phospholipid found exclusively in the inner mitochondrial membrane, is closely involved in maintaining a cristae structure and energy metabolism [20]. CL has a characteristic structure with four acyl chains. During maturation, one acyl chain of CL is enzymatically cleaved from immature CL, resulting in monolysocardiolipin (MLCL). MLCL is remodeled to mature CL primarily by the selective reattachment of linoleic acid [21]. In a fatty liver, the expression levels of genes involved in this remodeling pathway are reduced, leading to a decrease in mature CL and an increase in MLCL [22]. Increased MLCL is associated with mitochondrial dysfunction [23]. Thus, the decrease in MLCL contributes to mitochondrial energy metabolism. Further, lipid droplets and mitochondria are closely located and interact through the exchange of fatty acids [24]. Therefore, simultaneous analysis of the prevention of oxidized lipid droplet formation and the improvement of mitochondrial membrane quality may be useful for the multifaceted evaluation of antioxidants.

In this study, we developed a lipidomics-based cell screening platform to screen the preventive effects of indirect antioxidants on the formation of oxidized lipid droplets and the improvement of mitochondrial membrane quality simultaneously. C3A cells were pretreated with various antioxidants and stimulated with oxLDL to induce the formation of oxidized lipid droplets. To validate the lipidomics-based platform, we used representative flavonoids, kaempferol, quercetin, and hesperetin. Major lipids and their hydroperoxides were analyzed using lipidomics and fluorescence microscopy to evaluate their preventive effects on the formation of oxidized lipid droplets. CL and MLCL were analyzed using the same lipidomic data to evaluate the improvement of mitochondrial membrane quality. Finally, we also determined several specific lipid species that were effective in evaluating their preventive effects.

## 2. Materials and Methods

### 2.1. Separation and Oxidation of LDL

To prepare oxLDL, native LDL was isolated from human serum. Fasting serum samples were collected from healthy volunteers. As previously described [8], total lipoprotein fractions were recovered using ultracentrifugation, and the LDL fraction was purified using gel filtration high performance liquid chromatography (Shimadzu Corp., Kyoto, Japan) with a Superose 6 column (GE Healthcare, Little Chalfont, UK). The LDL protein concentration was measured using the Lowry method [25]. To prepare oxLDL, LDL was adjusted to 0.2 mg/mL and 1 mM CuSO_4_ was added to a final concentration of 0.06 mM. The mixture was incubated at 37 °C for 2 h. Thereafter, 31 mM EDTA·2Na was added to a final concentration of 1 mM to stop the oxidation. Cu^2+^ and EDTA were removed using a tube equipped with a 100 kDa ultrafiltration membrane filter (Merck Millipore Ltd., Cork, Ireland). Dulbecco’s PBS (D-PBS; FUJIFILM Wako Pure Chemical Industries Ltd., Osaka, Japan) was added to the sample to restore the original protein concentration, and the sample was centrifuged using a tube equipped with a 0.22 μm membrane filter (Merck Millipore Ltd.).

### 2.2. Cell Culture

A human liver-derived cell line, C3A (CRL-10741, American Type Culture Collection, Manassas, VA, USA) was used for cell culture experiments at passages 21 to 23. Cells were verified as mycoplasma-free at the time of purchase. Cells were cultured in Minimum Essential Medium (MEM; Thermo Fisher Scientific Inc., Waltham, MA, USA) containing 1% GlutaMAX supplemented with 10% fetal bovine serum (FBS; Biowest, Nuaillé, France) and 1% penicillin-streptomycin-neomycin (Thermo Fisher Scientific Inc.) at 37 °C under 5% CO_2_.

### 2.3. Cell Viability Test

Cell viability tests were performed using the WST-1 assay to determine optimal concentrations of oxLDL and antioxidants. Regarding oxLDL, C3A cells were seeded at 1.0 × 10^4^ cells/well in a 96-well plate at 100 µL/well and preincubated for 24 h. The culture medium was replaced with serum-free clear MEM (1% GlutaMAX, no FBS, no glutamine, and no phenol red; Thermo Fisher Scientific). After 24 h, the supernatant was removed and replaced with serum-free clear MEM containing oxLDL (up to 2 µg protein/mL, the maximum concentration obtainable from a fresh isolation) to develop lipid droplets or D-PBS as a control. After 22 h, the WST-1 reagent was prepared by mixing WST-1 (FUJIFILM Wako Pure Chemical Industries Ltd.) and 1-methoxy-phenazinemethosulfate (FUJIFILM Wako Pure Chemical Industries Ltd.) in a 9:1 ratio. Then, 50 µL of this reagent was added to the cells and incubated for another 2 h. The absorbance was measured at 450 nm using a microplate reader (Bio-Rad Laboratories, Hercules, CA, USA). Cell viability was expressed relative to that of control cells (n = 6).

C3A cells were seeded as described above. The culture medium was replaced with serum-free clear MEM (1% GlutaMAX, no FBS, no glutamine, no phenol red; Thermo Fisher Scientific) containing each antioxidant dissolved with ethanol (kaempferol, Cayman Chemical, Ann Arbor, MI, USA; quercetin, Sigma-Aldrich Chemicals, St. Louis, MO, USA; hesperetin, Cayman Chemical) at concentrations of up to 40 µM for kaempferol and hesperetin and 20 µM for quercetin, or ethanol alone as a control. After 24 h, the supernatant was removed, and the medium was replaced with serum-free MEM. As described above, cell viability tests were performed using WST-1 reagent.

### 2.4. Establishment of a Lipidomics-Based Cell Screening Platform for Oxidized Lipid Droplet Formation

We validated the concentration conditions described above to establish a lipidomics-based platform for investigating the preventive effects of various antioxidants against oxidized lipid droplet formation induced by oxLDL.

First, we confirmed that oxLDL induced oxidized lipid droplet formation using lipidomic analysis in a lipidomics-based platform. C3A cells were seeded at 1.0 × 10^5^ cells/well in a 24-well plate (1 mL/well) and preincubated for 24 h. Next, serum-free clear MEM containing ethanol (final concentration: 0.5%) was added to the cells. After 24 h, the supernatant was removed and replaced with serum-free clear MEM containing D-PBS (final concentration: 1%) as a control or oxLDL (2 µg protein/mL), and the cells were cultured for 24 h. The supernatant was then removed, and TryPLE (Thermo Fisher Scientific) (300 µL/well) was added to the cells for cell collection (3 wells = 1 sample, n = 6). Next, 300 µL of 10% FBS-containing clear MEM was added to each 1.5 mL tube, and the suspension was centrifuged (15,000× *g*, 4 °C, 5 min). The supernatant was then replaced with 500 µL of D-PBS. For protein concentration measurement, 50 µL of the suspension was collected and replaced with 50 µL of RIPA buffer to lyse the cells. The remaining 450 µL was collected as the cell pellet for lipidomic analysis. All samples were stored at −80 °C until analysis.

Using a lipidomics-based platform of oxidized lipid droplet formation and simultaneous lipid analysis, we investigated the preventive effects of various antioxidants. C3A cells were seeded as described above and treated for 24 h with serum-free clear MEM containing each antioxidant (final concentration: 5 µM) dissolved in ethanol (final concentration: 0.5%) or ethanol alone (0.5%) as a control. The oxLDL stimulation and cell collection were performed under identical conditions (n = 6 per group).

### 2.5. Extraction of Intracellular Lipids

Intracellular lipids were extracted using the Folch method [26]. A mixture of SPLASH LIPIDMIX Quantitative Mass Spec Internal Standard and CL56:0 (Avanti Polar Lipids, Inc., Alabaster, AL, USA) was prepared with methanol as internal standard (IS) for lipidomics [27,28]. Methanol (100 µL), 100 µL of IS, 400 µL of chloroform, and 100 µL of distilled water were added to the collected cell pellet. To increase the yield, the lipid extraction using 400 µL of chloroform was repeated. After evaporation, the extracts were redissolved in 100 µL of methanol. The supernatant was stored at −80 °C for lipidomic analysis.

### 2.6. Simultaneous Lipid Analysis Using Orbitrap Liquid Chromatography–Tandem Mass Spectrometry (LC-MS/MS)

The extracted intracellular lipids were analyzed using Orbitrap LC-MS/MS, as previously reported [27]. The target lipids for analysis were the hydroperoxides (-OOH) of triglycerides (TG), and cholesteryl esters (CE), which are abundant in lipid droplets. Furthermore, the hydroperoxides of phosphatidylcholine (PC) were analyzed to assess cellular oxidative stress [29]. CL and MLCL were also analyzed as lipids that constitute the inner mitochondrial membrane. A Shimadzu Prominence HPLC system (DGU-20A_3_, LC-20AD, SIL-20A, CTO-20A, Shimadzu Corp.) and an Atlantis T3 Column (C18, 2.1 × 150 nm, and 3 µm; Waters Corp., Milford, MA, USA) were used for the LC portion. The oven temperature was set at 40 °C, the flow rate was 200 µL/min, and the mobile phase consisted of 5 mM ammonium acetate solution, isopropanol, and methanol. The injection volume of each sample was 10 µL. An LTQ Orbitrap XL mass spectrometer (Thermo Fisher Scientific) was used to analyze the positive and negative ion modes with electrospray ionization. The peak areas of the target molecules were calculated from the mass spectra of each target lipid molecule and the IS using Xcalibur 2.2 (Thermo Fisher Scientific). The concentration of lipids was corrected for the peak area of IS, moles of added IS, and protein concentration. The protein concentrations were measured using the Pierce BCA Protein Assay Kit (Thermo Fisher Scientific).

### 2.7. Fluorescent Staining for Lipid Hydroperoxide

To visually estimate the formation of oxidized lipid droplets by oxLDL and the preventive effects of antioxidants, fluorescent staining for lipid hydroperoxide was conducted. C3A cells were seeded at 0.75 × 10^5^ cells/compartment in a four-compartment glass-bottom dish (Greiner Bio-One, Tokyo, Japan) at 0.5 mL/compartment and preincubated for 24 h. Stimulation was performed under the same conditions as those used for lipidomic analysis. After removing the supernatant and washing with D-PBS, 0.5 mL of staining solution was added to each compartment and incubated for 20 min at 37 °C under 5% CO_2_. The staining solution consisted of a 1000:1:3 mixture of serum-free clear MEM, Hoechst 33342 (nuclear stain, Fujifilm Wako Pure Chemical Corporation), and Liperfluo (Dojin Chemical Laboratories, Kumamoto, Japan). Liperfluo has high specificity for lipid peroxides [30]. After removing the staining solution and washing with D-PBS, the supernatant was replaced with FluoroBrite DMEM (Thermo Fisher Scientific), and cells were observed under a fluorescence microscope (HS all-in-one fluorescence microscope BZ-9000, Keyence Co., Ltd., Osaka, Japan) [31]. The excitation/emission wavelengths for Hoechst 33342 and Liperfluo were 352 nm/461 nm and 524 nm/535 nm, respectively. The exposure times for the Hoechst 33342, Liperfluo, and bright fields were 1/4 s, 2.8 s, and 1/50 s, respectively. Fluorescence images were analyzed using the HS all-in-one fluorescence microscope BZ-II analysis application (Keyence Co., Ltd.) and ImageJ 1.54g (NIH, Bethesda, MD, USA) to determine the area of the oxidized lipid droplets [32]. Cells with fully recognizable shapes were analyzed (76–104 cells per group). Oxidized lipid droplet circularity of 0.5–1.0 and area of 0.1–50 µm^2^ were estimated [33,34].

### 2.8. Statistical Analysis

Statistical analyses were performed using GraphPad Prism V7.0 software (GraphPad Software Inc., San Diego, CA, USA). After detecting outliers and performing rejection tests, normality was assessed, and either one-way ANOVA with Dunnett’s multiple comparisons test or Kruskal–Wallis test was used for comparisons among the three groups, and unpaired *t*-tests were used for comparisons between two groups. A *p*-value of 0.05 was considered statistically significant.

### 2.9. Ethical Approval

The study was conducted in accordance with the Declaration of Helsinki, and blood sampling to obtain LDL levels was conducted with approval of the Ethics Committee of the Faculty of Health Sciences, Hokkaido University (approval number: 19-107-5). Informed consent was obtained from all the participants.

## 3. Results

### 3.1. Oxidized Lipid Droplet Formation by oxLDL

**Figure 1:**
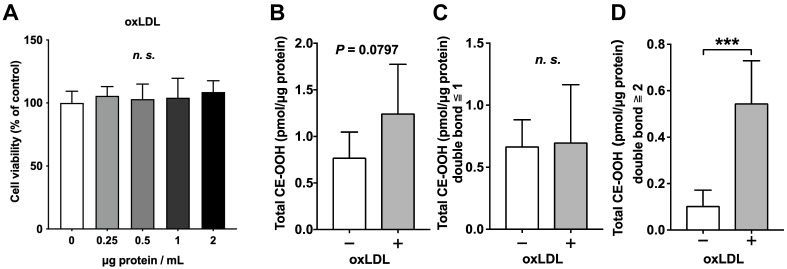
Establishment of a lipidomics-based cell screening platform on oxidized lipid droplet formation using Orbitrap LC-MS/MS. (**A**) Effects of oxLDL on cell viability in C3A cells. Cell viability of the control group (1% D-PBS-supplemented) was set as 100%. (**B**–**D**) CE-OOH species in oxLDL-supplemented cells. (**B**) Total CE-OOH. (**C**) Total CE-OOH with one or fewer double bonds. (**D**) Total CE-OOH with two or more double bonds. Total CE-OOH was determined as the sum of all CE-OOH species detected in this study. Results are expressed as means ± standard deviation. n = 6. One-way ANOVA with Dunnett’s multiple comparisons test, unpaired *t*-test, *** *p* < 0.001; *n.s.*, not significant. CE, cholesteryl ester; D-PBS, Dulbecco’s phosphate-buffered saline; oxLDL, oxidized low-density lipoprotein.

To determine the optimal oxLDL concentration, a cell viability test was performed in oxLDL-supplemented C3A cells. No significant changes in cell viability were observed within the concentration range tested in this study (Figure 1A). Thus, oxLDL at 2 µg protein/mL was used to induce oxidized lipid droplet formation in subsequent experiments.

To analyze oxidized lipid droplets, lipidomic analysis focused on TG, CE, TG-OOH, and CE-OOH. In this study, the amount of CE-OOH tended to be higher in the oxLDL group (control vs. oxLDL; *p* = 0.0797; Figure 1B). When CE-OOH species were classified according to the number of double bonds, no significant differences were observed between the control and oxLDL groups in CE-OOH with one or fewer double bonds (Figure 1C). In contrast, CE-OOH, which has two or more double bonds, significantly increased in the oxLDL group (Figure 1D). The levels of TG-OOH, PC-OOH, CE, TG, CL, and MLCL did not change in either the control or oxLDL groups (Supplementary Figure S1).

### 3.2. Cell Viability of Each Antioxidant

**Figure 2:**
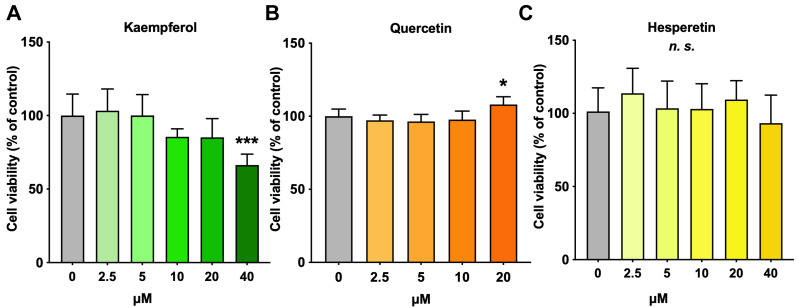
Effects of each antioxidant on cell viability in C3A cells. (**A**) Kaempferol, (**B**) quercetin, (**C**) hesperetin. Cell viability of the control group (0.5% ethanol-supplemented) was set as 100%. Results are expressed as means ± standard deviation. n = 6. One-way ANOVA with Dunnett’s multiple comparisons test, * *p* < 0.05; *** *p* < 0.001; *n.s.*, not significant.

To determine the optimal concentration of the various antioxidants, a cell viability test was performed for each antioxidant-supplemented C3A cell line. Kaempferol showed a decreasing trend at concentrations above 10 µM (Figure 2A). Quercetin and hesperetin did not significantly reduce cell viability at the tested concentrations (Figure 2B,C). Therefore, the antioxidant concentration used in this study was set at 5 µM.

### 3.3. Prevention of Oxidized Lipid Droplet Formation in Each Antioxidant-Supplemented C3A Cells

**Figure 3:**
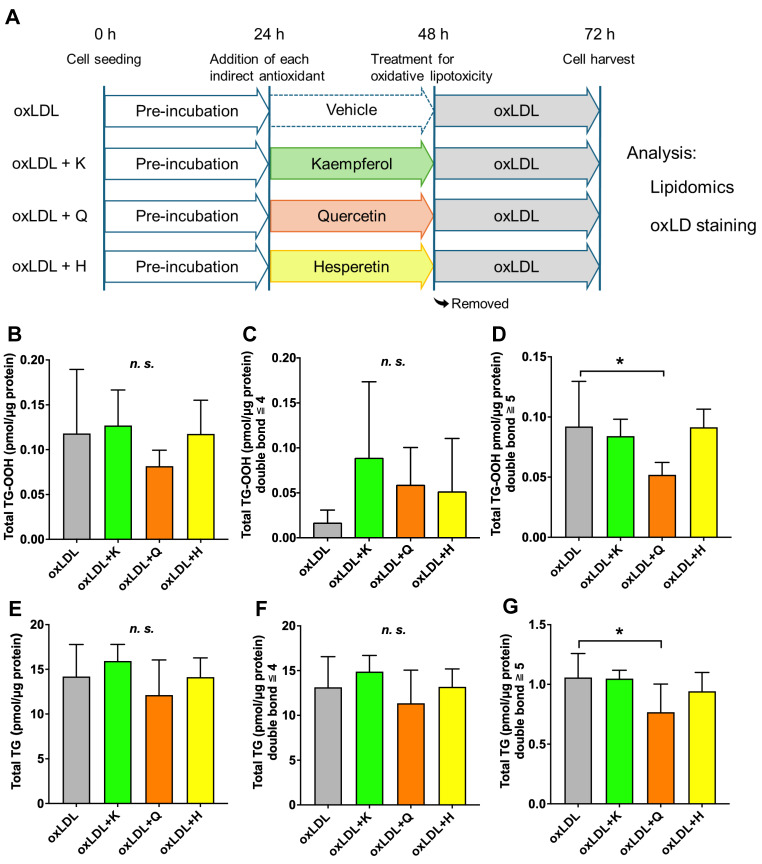
Preventive effect of various antioxidants against oxidized lipid droplet formation using oxLDL with simultaneous lipid analysis. (**A**) Schematic diagram of this lipidomics-based platform (**B**) Total TG-OOH, (**C**) Total TG-OOH with four or fewer double bonds, and (**D**) Total TG-OOH with five or more double bonds. (**E**) Total TG, (**F**) Total TG with four or fewer double bonds, and (**G**) Total TG with five or more double bonds. Total TG-OOH and TG were determined as the sum of all species detected in this study. Results are expressed as means ± standard deviation. n = 6. One-way ANOVA with Dunnett’s multiple comparisons test, * *p* < 0.05, *n.s.* not significant. oxLDL, oxidized low-density lipoprotein; TG, triglyceride.

This lipidomics-based analysis was carried out as follows: First, C3A cells were seeded and treated. Then, C3A cells were treated for 24 h with the antioxidant for pre-treatment against oxLDL-driven lipotoxicity. Then, the antioxidant was removed, and oxLDL was added to form oxidized lipid droplets for 24 h. After that, we analyzed lipids such as lipid hydroperoxides and mitochondrial membrane lipids. Furthermore, fluorescence microscopy was performed to verify the efficacy of antioxidants against oxidized lipid droplets (Figure 3A). Eleven TG-OOH species were detected in the oxLDL-supplemented C3A cells (Supplementary Figure S2A). No significant difference was noted in the amount of TG-OOH in the C3A cells when each antioxidant was added (Figure 3B). Similarly, no significant changes occurred in the amount of TG-OOH with four or fewer double bonds among the antioxidants (Figure 3C). However, the amount of TG-OOH with five or more double bonds significantly decreased only in the quercetin group compared to that in the oxLDL group (Figure 3D). Fifty-nine TG species were detected in oxLDL-supplemented C3A cells (Supplementary Figure S2B). Similar to TG-OOH, the addition of each antioxidant did not significantly change the total amount of TG or TG with four or fewer double bonds (Figure 3E,F). In contrast, the amount of TG with five or more double bonds was significantly decreased only in the quercetin group compared with that in the oxLDL group (Figure 3G).

**Figure 4:**
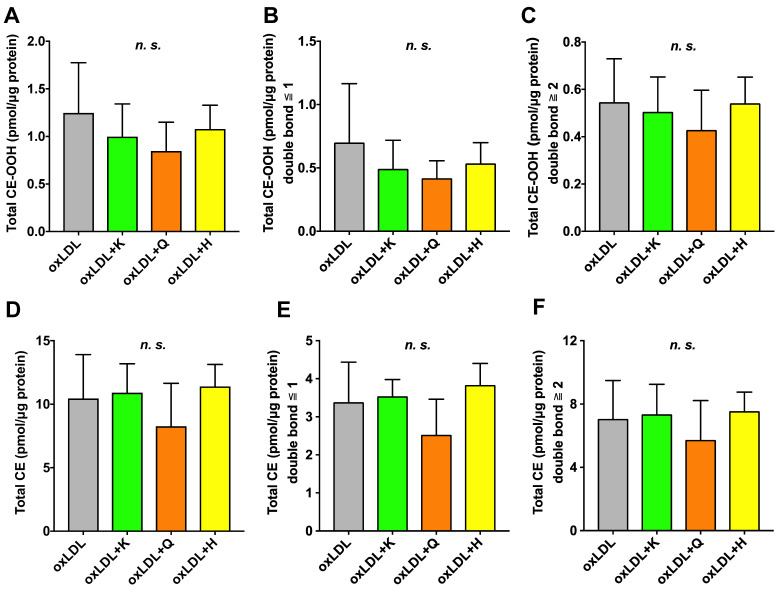
Preventive effects of various antioxidants against oxidized lipid droplet formation induced by oxLDL with simultaneous lipid analysis. (**A**) Total CE-OOH, (**B**) Total CE-OOH with one or fewer double bonds, and (**C**) Total CE-OOH with two or more double bonds. (**D**) Total CE, (**E**) Total CE with one or fewer double bonds, and (**F**) Total CE with two or more double bonds. Total CE-OOH and CE were determined as the sum of all species detected in this study. Results are expressed as means ± standard deviation. n = 6. One-way ANOVA with Dunnett’s multiple comparisons test, *n.s*., not significant. oxLDL, oxidized low-density lipoprotein; K, kaempferol; Q, quercetin; H, hesperetin; CE, cholesteryl ester; CE-OOH, cholesteryl ester hydroperoxides.

In the case of CE-OOH, three CE-OOH species were detected in oxLDL-supplemented C3A cells (Supplementary Figure S2C). The total amounts of CE-OOH, CE-OOH with one or fewer, and CE-OOH with two or more double bonds did not exhibit significant differences in any of the antioxidant-supplemented C3A cells (Figure 4A–C). Similarly, 11 CE species were detected in oxLDL-supplemented C3A cells (Supplementary Figure S2D), and the amount of CE remained unchanged in all groups (Figure 4D–F).

**Figure 5:**
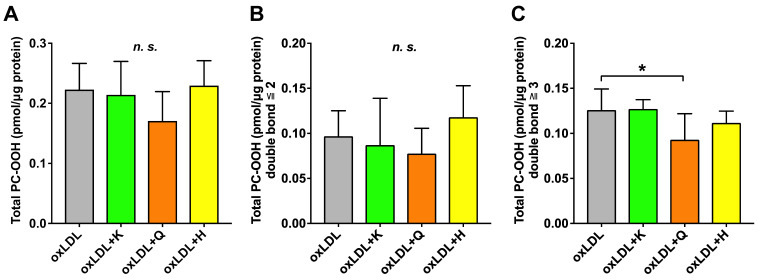
Preventive effects of various antioxidants against oxidized lipid droplet formation induced by oxLDL with simultaneous lipid analysis. (**A**) Total PC-OOH, (**B**) Total PC-OOH with two or fewer double bonds, and (**C**) Total PC-OOH with three or more double bonds. Total PC-OOH was determined as the sum of all species detected in this study. Results are expressed as means ± standard deviation. n = 6. One-way ANOVA with Dunnett’s multiple comparisons test, * *p* < 0.05; *n.s*., not significant. oxLDL, oxidized low-density lipoprotein; K, kaempferol; Q, quercetin; H, hesperetin; PC-OOH, phosphatidylcholine hydroperoxides.

Six PC-OOH species were detected in oxLDL-supplemented C3A cells (Supplementary Figure S2E). The total amount of PC-OOH did not significantly change in any of the antioxidant-supplemented C3A cells (Figure 5A). In addition, no significant changes were noted in the amount of PC-OOH with two or fewer double bonds in any group (Figure 5B). In contrast, the amount of PC-OOH with three or more double bonds significantly decreased only in the quercetin group compared with that in the oxLDL group (Figure 5C).

### 3.4. Evaluation of Oxidized Lipid Droplets Using Fluorescence Microscopy

**Figure 6:**
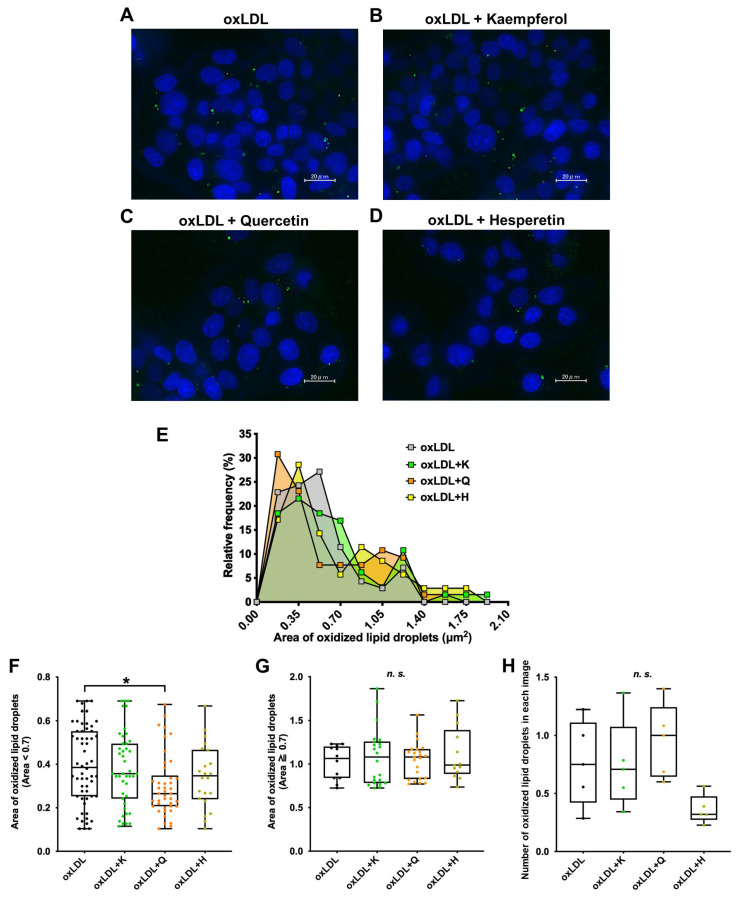
Preventive effects of various antioxidants against oxidized lipid droplet formation induced by oxLDL with fluorescence microscopy. (**A**–**D**) Oxidized lipid droplets formed by C3A cells supplemented with (**A**) oxLDL, (**B**) oxLDL + kaempferol, (**C**) oxLDL + quercetin, and (**D**) oxLDL + hesperetin. Blue: nuclei, green: oxidized lipid droplets. (**E**) Histogram of the area of oxidized lipid droplets. (**F**) Oxidized lipid droplets with areas of less than 0.7 µm^2^. (**G**) Oxidized lipid droplets with areas of more than 0.7 µm^2^. (**H**) Number of oxidized lipid droplets in each image. Results are expressed as a box plot (n = 76–104). One-way ANOVA with Kruskal–Wallis test, * *p* < 0.05; *n.s.*, not significant. oxLDL, oxidized low-density lipoprotein; K, kaempferol; Q, quercetin; H, hesperetin.

The oxLDL-induced formation of oxidized lipid droplets and the preventive effect of each antioxidant were evaluated using fluorescent images (Figure 6A–D). The area of the oxidized lipid droplets, which should be related to the results of the comprehensive lipid analysis using mass spectrometry, was drawn using a histogram to visualize the size distribution of oxidized lipid droplets (Figure 6E). In the present study, the peak areas of the most frequent oxidized lipid droplets in oxLDL, oxLDL + kaempferol, oxLDL + quercetin, and oxLDL + hesperetin were 0.525, 0.35, 0.175, and 0.35 µm^2^, respectively (Figure 6E). A small second peak corresponding to large oxidized lipid droplets was observed at 0.875–1.225 µm^2^.

Next, we divided the oxidized lipid droplets into two groups based on size and compared the two groups. Oxidized lipid droplets < 0.7 µm^2^ were defined as small droplets, and those ≥0.7 µm^2^ were defined as large droplets (Figure 6F,G). The size of the small oxidized lipid droplets was significantly decreased only in the quercetin group compared to that in the oxLDL group. In contrast, no significant change was observed in the size of large oxidized lipid droplets in any group. No significant changes in oxidized lipid droplet size were observed in the kaempferol or hesperetin groups compared with those in the oxLDL group.

Furthermore, we analyzed the number of oxidized lipid droplets (Figure 6H). A decreasing trend in the number of oxidized lipid droplets was observed in the hesperetin-treated group.

### 3.5. Effect of Antioxidants on Improving Mitochondrial Membrane Lipids

**Figure 7:**
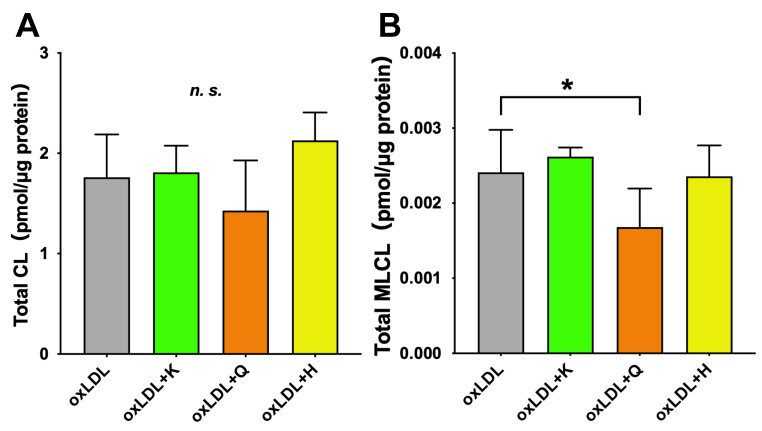
Effect of antioxidants on improving mitochondrial membrane lipids. (**A**) Total CL, (**B**) Total MLCL. Total CL and total MLCL were determined as the sum of all species detected in this study, respectively. Results are expressed as means ± standard deviation. n = 6. One-way ANOVA with Dunnett’s multiple comparisons test, * *p* < 0.05; *n.s*., not significant. oxLDL, oxidized low-density lipoprotein; CL, cardiolipin; MLCL, monolysocardiolipin.

Twenty-four CL and nine MLCL species were detected in oxLDL-supplemented C3A cells (Supplementary Figure S2F,G). The addition of antioxidants did not change the total amount of CL (Figure 7A).

The total amount of MLCL significantly decreased only in the quercetin group (Figure 7B). However, no significant changes were observed in the kaempferol and hesperetin groups.

**Figure 8:**
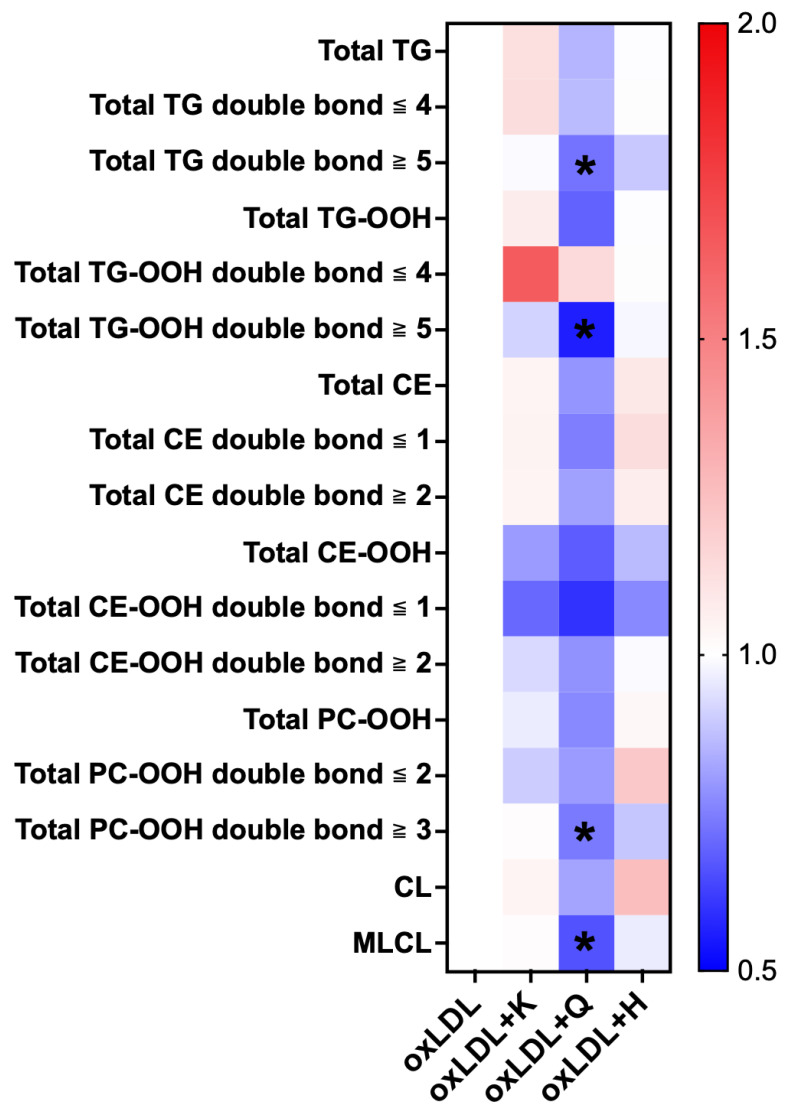
Heat map of each lipid in the C3A cells supplemented with oxLDL. Regarding color intensities, the mean values of each lipid in the oxLDL group are expressed as 1.0. Individual results for the samples are represented (n = 6 for each group). The results are expressed as means ± standard deviation. One-way ANOVA with Dunnett’s multiple comparisons test, * *p* < 0.05 vs. oxLDL group. oxLDL, oxidized low-density lipoprotein; K, kaempferol; Q, quercetin; H, hesperetin; TG, triglyceride; TG-OOH, triglyceride hydroperoxides; CE, cholesteryl ester; CE-OOH, cholesteryl ester hydroperoxides; PC-OOH, phosphatidylcholine hydroperoxides; CL, cardiolipin; MLCL, monolysocardiolipin.

As shown in the heat map (Figure 8), the levels of various lipid species were lower in the quercetin group than in the oxLDL group. In particular, TG with five or more double bonds, TG-OOH with five or more double bonds, and MLCL significantly decreased in the quercetin group.

## 4. Discussion

We used oxidized LDL to verify the induction of oxidized lipid droplet formation in C3A cells. Fluorescence microscopy revealed the presence of oxidized lipid droplets in C3A cells treated with oxLDL. Furthermore, LC-MS/MS revealed that the cells showed an increase in the amount of CE-OOH, which has two or more double bonds, including CE-OOH 18:2. A previous study showed that oxLDL prepared under the same conditions as those in the present study contained a large amount of CE-OOH, especially CE-OOH 18:2 [8], which is consistent with our results. Therefore, the uptake of oxLDL into C3A cells could induce oxidized lipid droplet formation. In contrast, TG-OOH and PC-OOH are present, but their levels are not increased. This likely reflects the reality of the phenotype in oxLDL-driven oxidative lipotoxicity. The types of lipid peroxides reduced by antioxidants may differ, and we believe that comparing their effects using this platform will create scientific novelty.

To investigate the preventive effects of various antioxidants on oxidized lipid droplet formation, three flavonoids were used in the proposed lipidomics-based platform. In fluorescence microscopy analysis, a decreasing trend in the number of oxidized lipid droplets was observed in the hesperetin-treated group. However, while mass spectrometry is highly sensitive and capable of detecting lipid hydroperoxide species, many cells lacked oxidized lipid droplets in the fluorescent staining method, which suggests that the number of oxidized lipid droplets was below the limit of detection. Therefore, analyzing the number was not suitable for this fluorescent microscopy method. On the other hand, the value of the area should be related to the results of the comprehensive lipid analysis using mass spectrometry, which is a feature of this study. Thus, we consider that the area value was more appropriate in this study. Interestingly, fluorescence microscopy also revealed that quercetin reduced the area of oxidized lipid droplets. This finding is consistent with previous reports wherein lipid droplet regression was observed in the livers of mice fed a high-fat diet containing quercetin or in HepG2 cells loaded with palmitic and oleic acids [35,36]. Additionally, a key feature of our proposed method is the ability to perform detailed lipidomic analyses using mass spectrometry. Although quercetin is a well-known antioxidant, to the best of our knowledge, this is the first study to demonstrate that quercetin significantly decreases not only major lipids but also lipid hydroperoxide species, particularly TG and TG-OOH, with five or more double bonds. This decrease may be attributable to quercetin-induced degradation of these lipids. This degradation is thought to be mediated by ATGL, an enzyme that hydrolyzes TG. This hypothesis is consistent with reports that at concentrations higher than 5 µM, quercetin enhances ATGL expression [37]. Furthermore, quercetin decreased levels of PC-OOH species with multiple double bonds and showed a decreasing trend in CE-OOH species with many double bonds, which may be explained by the reduction in lipid hydroperoxides by quercetin. Glutathione peroxidase 4 (GPx4) is an antioxidant enzyme induced via the Nrf2 pathway that reduces lipid hydroperoxides in biological membranes to lipid alcohols [38]. GPx4 can use PC-OOH and CE-OOH, the major lipids in lipid droplets, as substrates [39,40]. Quercetin at 10 µM activates the Keap1-Nrf2 pathway and reduces lipid hydroperoxides via GPx4 [41]. The reduction in the amount of CE-OOH was less than that of PC-OOH. This suggests that GPx4 preferentially binds to the polar sites of phospholipids [42]; therefore, its antioxidant effect on the non-polar lipid CE-OOH is limited. Increased MLCL levels are associated with mitochondrial dysfunction [23]. MLCL also leads to insufficient ATP production via the electron transport chain [43]. Decreased MLCL levels could be associated with increased ATP production [27]. Therefore, it is speculated that the reduction in total MLCL levels by quercetin in this model contributed to the improvement of mitochondrial cristae structure and function. This is also consistent with a previous report showing that quercetin inhibited the decline in mitochondrial membrane potential and significantly increased ATP production in oleic acid-induced hepatoma cells, HepG2 [44].

The maximum blood concentrations of the three flavonoids reported after oral intake in humans are as follows: kaempferol, 0.10 µM (36.9 mg kaempferol/300 g portion as endive soup); quercetin, 0.43 µM (150 mg quercetin as a supplement); and hesperetin, 0.10 µM (71.8 mg hesperetin [as hesperidin]/300 g portion as orange juice) [15,17,45], which are lower than that in the present study. For example, in kaempferol, assuming that the total blood volume of a typical adult is approximately 5 L, the absorption rate into the blood is therefore estimated as 0.39%, which means low bioavailability. Therefore, the 5 µM concentration used in this study cannot be achieved through oral intake. However, we consider the use of an *in vitro* lipidomics-based platform to be necessary and justified for several reasons. First, lipidomics-based platforms allow analysis of the direct, cell-autonomous effects of polyphenols without confounding *in vivo* factors, such as metabolism and tissue distribution. This reduction in complexity is essential for identifying key molecular targets, signaling pathways, and dose–response relationships. Second, because many polyphenols undergo extensive biotransformation *in vivo*, elucidating their intrinsic cellular activities provides an important mechanistic foundation for interpreting more complex *in vivo* findings. Third, lipidomics-based platforms permit controlled exposure to defined concentrations and time frames that cannot be achieved with sufficient precision in animal or human studies. Moreover, our established cell assays recapitulate key physiological features relevant to the biological functions examined, including oxidative stress responses, mitochondrial function, and lipid metabolism. The reproducibility and sensitivity of the cell-based system make it suitable for screening and mechanistic investigations, which are essential preliminary steps before conducting *in vivo* experiments. Therefore, we believe that the use of this lipidomics-based platform is scientifically justified as an important mechanistic approach for comprehensively evaluating the functionality of polyphenols.

Nonetheless, this study has some limitations. We used a model in which oxidized lipid droplet formation was prevented by adding indirect antioxidants before oxLDL stimulation. This is a pre-treatment for acute stimulation *in vitro* and does not fully reflect the long-term preventive effect *in vivo*. To determine the actual preventive effect, validation of the *in vivo* experiments is required following the screening in this study, which should motivate future *in vivo* studies. A previous investigation revealed that a 98-day toxicity study showed that quercetin did not cause adverse effects in mice at doses where its efficacy was demonstrated. In particular, no increase in liver function markers (aspartate aminotransferase, alanine aminotransferase) was observed [46]. Also, focusing on changes in ATP and cristae structure, and experiments using primary hepatocytes/organoid models could lead to the expansion of this platform.

## 5. Conclusions

A lipidomics-based platform was established to screen antioxidants to prevent the formation of oxidized lipid droplets and CL abnormality. Comprehensive lipid analysis using mass spectrometry revealed that targeting the TG-OOH molecular species with many double bonds is useful for evaluating the preventive effects of antioxidants on oxidized lipid droplet formation, and that targeting MLCL is useful for evaluating the effects of improving mitochondrial membrane quality. We demonstrated that, using antioxidants such as quercetin, this method offers value in detecting antioxidants with excellent preventive effects against TG-OOH and MLCL.

Our proposed platform could be applied to discovering the novel efficacy of antioxidants at the maximum non-toxic doses and non-toxic doses relevant to *in vivo* conditions. Second, their evaluation in this platform could lead to *in vivo* and mechanistic studies with optimal doses and duration, which should be performed in future human studies.

## Data Availability

Data for this article are available at Open Science Framework at https://osf.io/nadqb/overview?view_only=2cf5a2e19d0c4364945617aec9f32988, accessed on 20 February 2026.

## Supplementary Materials

The following supporting information can be downloaded at: https://www.mdpi.com/article/10.3390/nu18050719/s1, Figure S1: Comparison of each lipid species in oxLDL-supplemented cells; Figure S2: Comparison of each lipid species in the C3A cells supplemented with oxLDL and each indirect antioxidant detected using Orbitrap LC-MS/MS.

## Abbreviations

The following abbreviations are used in this manuscript:

CL	cardiolipin
MASLD	metabolic dysfunction-associated steatotic liver disease
oxLDL	oxidized low-density lipoprotein
NAFLD/NASH	nonalcoholic fatty liver disease/hepatitis
MLCL	monolysocardiolipin
D-PBS	Dulbecco’s PBS
FBS	fetal bovine serum
IS	internal standard
TG	triglycerides
CE	cholesteryl esters
PC	phosphatidylcholine
LC-MS/MS	liquid chromatography-tandem mass spectrometry
GPx4	glutathione peroxidase 4
