# Supplementary material for: Development of a Lipidomics-Based Cell Screening Platform for Indirect Antioxidants Targeting Oxidized Lipid Droplet Formation and Mitochondrial Membrane Abnormality
Source: Nutrients. 2026 Feb 24;18(5):719. doi: 10.3390/nu18050719 (PMC12986757; doi:10.3390/nu18050719)
Supplement: Supplementary file 1 [file nutrients-18-00719-s001.zip › nutrients-4135743-supplementary.pdf]

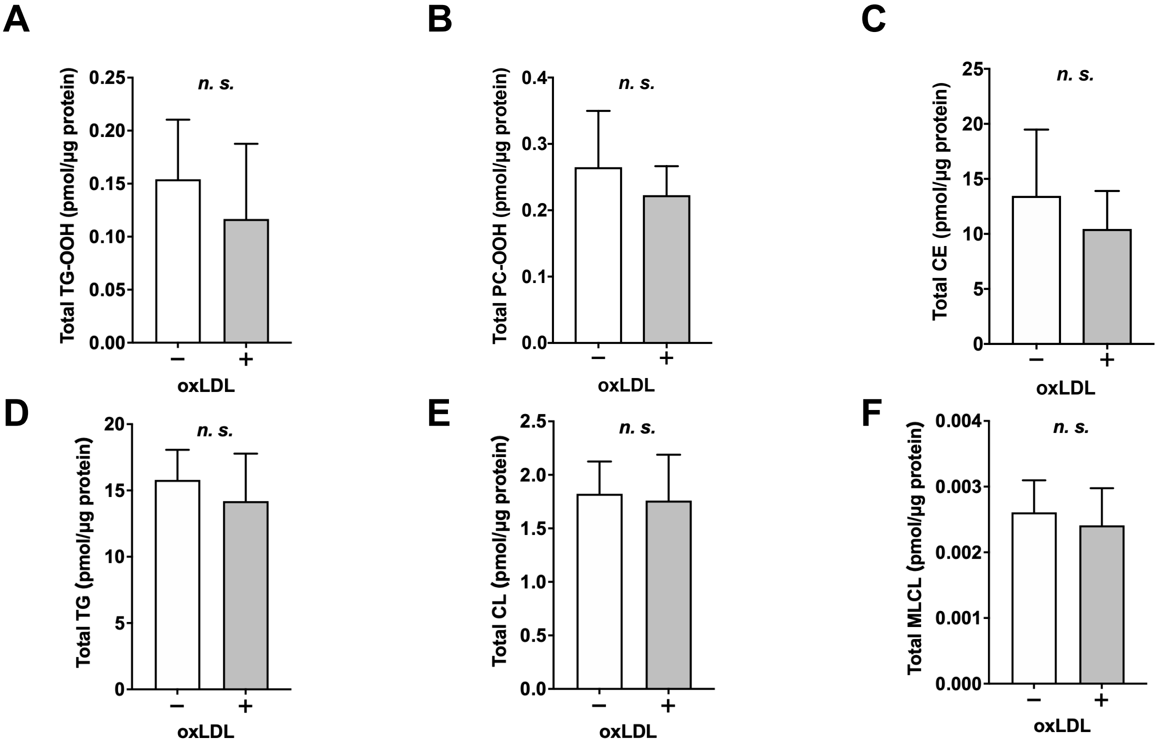

Supplementary figure S1. Comparison of each lipid species in oxLDL-supplemented cells. (A) Total TG-OOH, (B) Total PC-OOH, (C) Total TG, (D) Total CE, (E) Total CL, and (F) Total MLCL. Total TG-OOH, PC-OOH, CE, TG, CL and MLCL were determined as the sum of each species detected in this study. Results are expressed as means ± standard deviation. n = 6. Unpaired t-test, *n.s.*, not significant. oxLDL, oxidized low-density lipoprotein; TG-OOH, hydroperoxides; PC-OOH, phosphatidylcholine hydroperoxides; CE, cholesteryl ester; TG, triglyceride; CL, cardiolipin; MLCL, monolysocardiolipin.

A

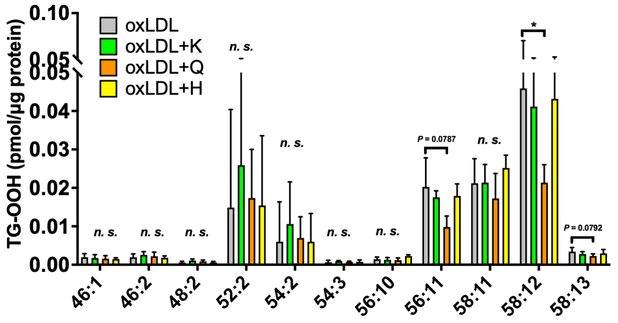
oxLDL
oxLDL+K
oxLDL+Q
oxLDL+H
TG-OOH (pmol/μg protein)
46:1
46:2
48:2
52:2
54:2
54:3
56:10
56:11
58:11
58:12
58:13
n. s.
P = 0.0787
P = 0.0792
*

B

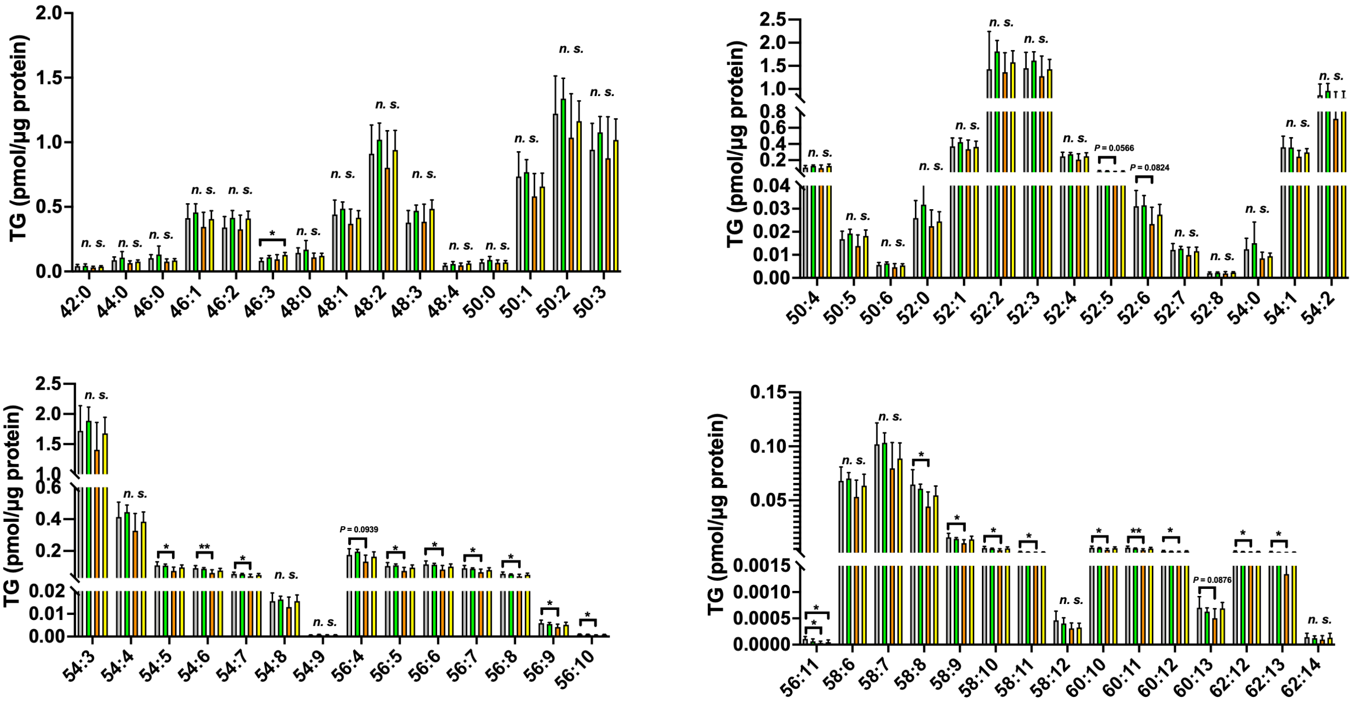
TG (pmol/μg protein)
42:0
44:0
46:0
46:1
46:2
46:3
48:0
48:1
48:2
48:3
48:4
50:0
50:1
50:2
50:3
50:4
50:5
50:6
52:0
52:1
52:2
52:3
52:4
52:5
52:6
52:7
52:8
54:0
54:1
54:2
P = 0.0566
P = 0.0824
54:3
54:4
54:5
54:6
54:7
54:8
54:9
56:4
56:5
56:6
56:7
56:8
56:9
56:10
P = 0.0939
56:11
58:6
58:7
58:8
58:9
58:10
58:11
58:12
60:10
60:11
60:12
60:13
62:12
62:13
62:14
P = 0.0876
n. s.
*
**

(Continued)

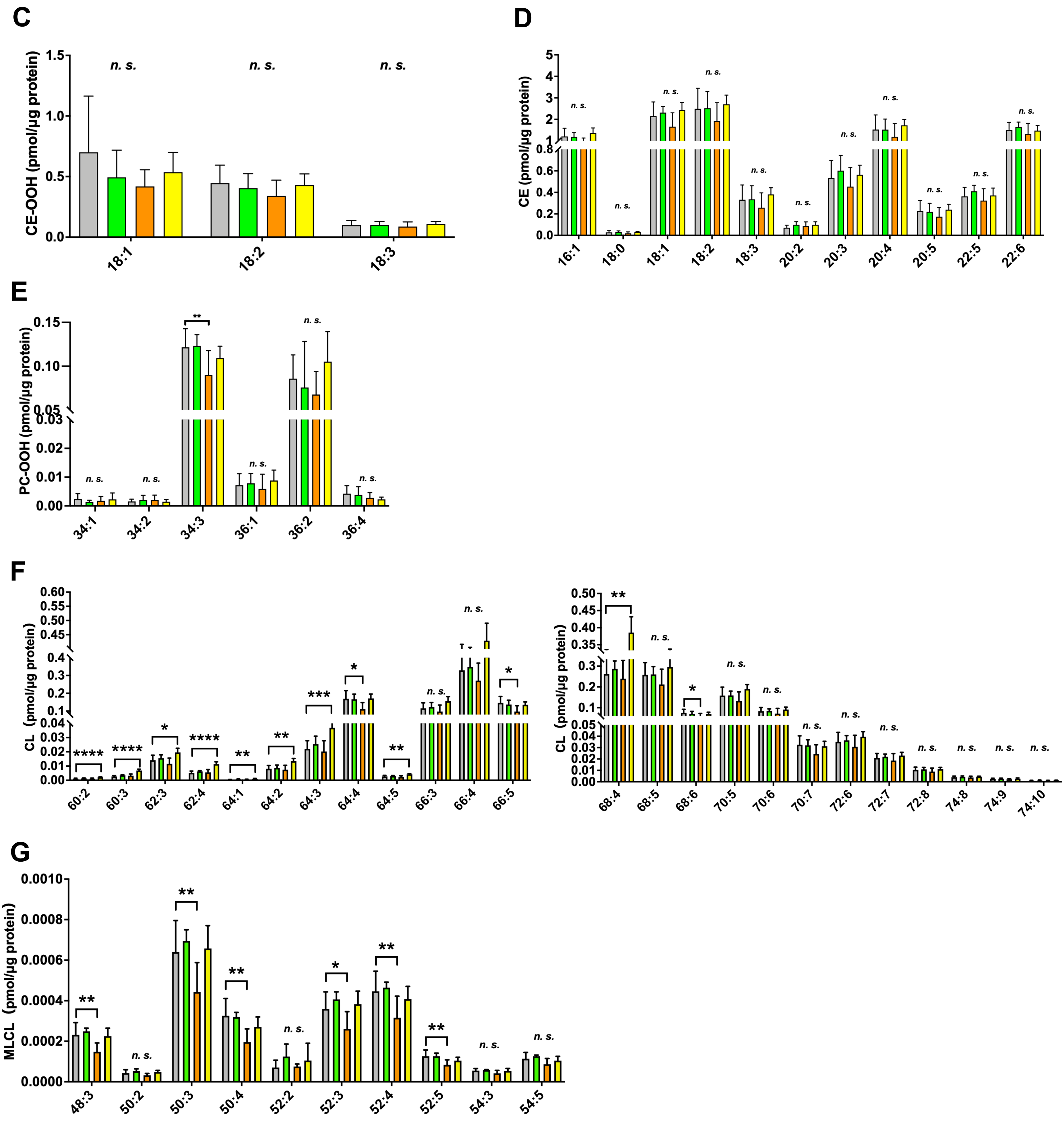

Supplementary figure S2. Comparison of each lipid species in the C3A cells supplemented with oxLDL and each indirect antioxidant detected using Orbitrap LC-MS/MS. (A) TG-OOH profile, (B) TG profile, (C) CE-OOH profile, (D) CE profile, (E) PC-OOH profile, (F) CL profile, and (G) MLCL profile. Results are expressed as mean ± standard deviation. n = 6. One-way ANOVA with Dunnet's multiple comparisons test, *$P < 0.05$, ** $P < 0.01$, *** $P < 0.001$, *n.s.* not significant. oxLDL, oxidized low-density lipoprotein; TG, triglyceride; TG-OOH, TG hydroperoxides; CE, cholesteryl ester; CE-OOH, CE hydroperoxides; PC-OOH, phosphatidylcholine hydroperoxides; CL, cardiolipin; MLCL, monolysocardiolipin.
